# Outcomes of Vertical Split Conjunctival Autograft Using Fibrin Glue in Treatment of Primary Double-Headed Pterygia

**DOI:** 10.1155/2018/9341846

**Published:** 2018-12-20

**Authors:** Tarek Roshdy Elhamaky, Ahmed Mohammed Elbarky

**Affiliations:** ^1^Ophthalmology Department, Lecturer of Ophthalmology, Benha University Faculty of Medicine, Benha 13511, Qalyubia Governorate, Egypt; ^2^Consultant Ophthalmologist, Advanced Center for Day Care Surgery, Abu Dhabi, UAE; ^3^Ophthalmology Department, Associate Professor Ophthalmology, Benha University Faculty of Medicine, Egypt; ^4^Consultant Vitreoretinal, Sheikh Khalifa Medical City, Abu Dhabi, UAE

## Abstract

**Purpose:**

To evaluate the efficiency of pterygium excision with the vertical split conjunctival technique using fibrin glue in treatment of primary double-headed pterygia.

**Patients and Methods:**

15 eyes of 15 patients with primary double-headed pterygia that underwent vertical split conjunctival autograft pterygium surgery were retrospectively reviewed. Recurrence was defined as fibrovascular proliferation over the limbus onto the cornea.

**Results:**

The patients' mean age was 36.92 ± 10.8 years. At 12-month follow-up, recurrence was not seen in any cases. Regarding postoperative cosmetic grading, grade 1 (the appearance of the operated site is not different from the normal appearance) was found in 12 eyes (80%) and grade 2 (some fine episcleral vessels in the excised area extending up to but not beyond the limbus and without fibrous tissue) was found in 3 eyes (20%). None of the cases showed conjunctival scarring or fibrosis at the conjunctival donor area. Preoperative Sim K astigmatism at the central 3 mm and BCVA were 3.05 ± 1.5 diopters (D) and 0.64 ± 0.26 logMAR, which improved significantly to 1.15 ± 0.84 D and 0.26 ± 0.18 logMAR at 12-month follow-up postoperatively, respectively.

**Conclusion:**

Vertical split conjunctival autograft using fibrin glue is an effective technique with good cosmetic results and low to no recurrence for primary double-headed pterygia treatment. This trial is registered with NCT03507283.

## 1. Introduction

Pterygium is a common ocular surface disease characterized by the invasion of fibrovascular tissue from the bulbar conjunctiva onto the cornea [1].

Ultraviolet (UV) exposure is a main cause of the formation of pterygia [2].

Histopathologically, pterygia are characterized by a hyperplasia of altered limbal epithelial cells, Bowman's layer dissolution, epithelial-mesenchymal transition, and stromal fibroblastic activation with neovascularization, inflammation, and matrix remodeling, mediated by cytokines, growth factors, and matrix metalloproteinases [3–5].

Some others suggested an abnormal tear function is a risk factor for pterygium formation, while others considered it as a sequela of changes involving pterygia [6, 7].

It is common to encounter pterygia in the “pterygium belt” region, which is located between 30° north and 30° south of the equator [8]. The pterygium usually commences at the nasal limbus (97%). A minority of pterygia is double-headed (with both temporal and nasal origins), and an isolated temporal pterygium is very rare [9]. The propensity for nasal pterygium is due to the peripheral UV light striking the eye laterally focused onto the medial limbal region [10]. Efforts should be made to differentiate temporal pterygia from squamous cell neoplasms [11].

Pterygium surgery is indicated mainly for visual impairment and cosmetic disfigurement and rarely for changes suggestive of neoplasia [12]. Surgical removal is the treatment of choice. The main challenge of pterygium excision is the avoidance of recurrence [13]. Conjunctival autografting is the best method, giving both a low recurrence rate and fewer side effects [14].

Tisseel (Baxter, Vienna, Austria) is a two-component tissue adhesive mimicking the physiologic wound healing process [15].

Double-headed pterygia present the surgeon with some unique problems including whether nasal and temporal pterygia on one eye should be dealt with simultaneously or separately, and the best method to deal with each pterygium remains uncertain [16].

We carried out a retrospective study to assess the efficiency of vertical split conjunctival autografting using fibrin glue in treatment of primary double-headed pterygia.

## 2. Patients and Methods

### 2.1. Patient Selection and Data Collection

A retrospective study was performed on 15 eyes of 15 patients that underwent vertical split conjunctival autograft pterygium surgery between April 2011 and June 2016. All patients had primary double-headed pterygia. All operations were performed by one surgeon (T. E.).

Baseline preoperative examination and all subsequent follow-up visits at 1 day, 1 week, and 1, 3, and 12 months included recording of best corrected visual acuity (BCVA) by the Snellen chart, slit-lamp biomicroscopy, autorefraction/autokeratometry (KR-8900 Autorefractor Keratometer; Topcon Corporation, Tokyo, Japan), computerized corneal topography (Pentacam HR; Oculus Optikgeräte GmbH, Wetzlar, Germany), tear film breakup time (BUT), Schirmer's test I, pterygium grade, pterygium morphology, pterygium size (which was measured from the limbus to the head of the pterygium), operative time (which was calculated using the stopwatch from the time of speculum insertion to speculum removal), conjunctival defects and grafts size, and postoperative cosmetic grading and recurrence.

Corneal photographs were taken by SL cam 5.0 (fully integrated and 5 megapixels) attached to the slit-lamp microscope (Zeiss SL 220; Carl Zeiss Meditec AG, Jena, Germany).

Pterygium was graded depending on the extent of its head onto the cornea [17]:

- Grade I: crossing the limbus
- Grade II: midway between the limbus and pupil
- Grade III: reaching up to the pupillary margin
- Grade IV: crossing the pupillary margin

Pterygium morphology was graded according to Tan et al. into atrophic (T1), intermediate (T2), or fleshy (T3) [18].

### 2.2. Surgical Technique

After instillation of topical proparacaine 0.5% (Alcaine; Alcon Canada, Couvreur, Belgium), the involved eye underwent sterile preparation and draping, and then a Barraquer lid speculum was inserted. The bodies of the nasal and temporal pterygia were marked. Nasal pterygia were operated on first as follows: 0.5 ml of lidocaine 2% with 1 : 10000 epinephrine was injected into the pterygium head. The pterygium was dissected from the cornea in a plane deep to Bowman's layer starting at 1 mm proximal to the pterygium head by the No. 15 Bard-Parker blade. The pterygium body was dissected from the sclera. Complete excision of the pterygium, subconjunctival Tenon's tissue, and surrounding fibrovascular tissues was followed with Westcott scissors. Light cautery was applied to sclera bleeding points if needed. The conjunctival edge was trimmed. The corneal surface was gently scraped with a scalpel. The same procedure was done on temporal pterygia. The conjunctival defects were measured by Castroviejo calipers. A free conjunctival graft of the same size of both nasal and temporal conjunctival defects was obtained from the superior bulbar conjunctiva. The conjunctiva was inflated with 0.5 ml lidocaine 2% with 1 : 10000 epinephrine solution and then dissected from Tenon's capsule toward the cornea. The graft was divided vertically into two parts: the nasal part of the graft was cut from the limbal attachment including limbal tissue and the temporal part was left attached. The nasal part of the graft was moved to the nasal conjunctival defect and attached to the sclera with the fibrin sealant (Tisseel; Baxter, Vienna, Austria). The same procedure was done with the temporal part of the graft. The limbal orientation of the graft towards the cornea and the epithelial side up was ensured before securing it. The donor site was closed with fibrin glue. Antibiotic steroid ointment was applied to the operated eye, and a pressure patch was kept in place for 24 hours.

Postoperatively, the patients were treated with tobramycin-dexamethasone and sodium hyaluronate 0.2% eye drops four times a day for 1 month.

### 2.3. Recurrence and Cosmetic Grading

The cosmetic appearance was graded from 1 to 4: in grade 1, the appearance of the operated site is not different from the normal appearance; grade 2 represents some fine episcleral vessels in the excised area extending up to but not beyond the limbus and without fibrous tissue; grade 3 includes more fibrous tissue in the excised area but does not invade the cornea; and grade 4 indicates a true recurrence with fibrovascular tissue invading the cornea [19].

### 2.4. Study Approval

The study was approved by the local ethics committee, and all patients signed an informed consent. This study has followed the tenets of the Declaration of Helsinki. ClinicalTrials.gov ID of this study is NCT03507283.

### 2.5. Statistical Analysis

Variables were expressed as percentage or mean ± standard deviation. Visual acuity was expressed as logMAR (logarithm of the minimum angle of resolution) for statistical analysis. To analyze changes in variables, paired Student's *t*-test was used. A *P* value <0.05 was considered statistically significant. The statistical significance was defined at 95% confidence intervals. Statistical analysis was performed using SPSS software version 16.0 for Windows (SPSS, Inc., Chicago, IL).

### 2.6. Primary Outcome Measures

**Figure 1:**
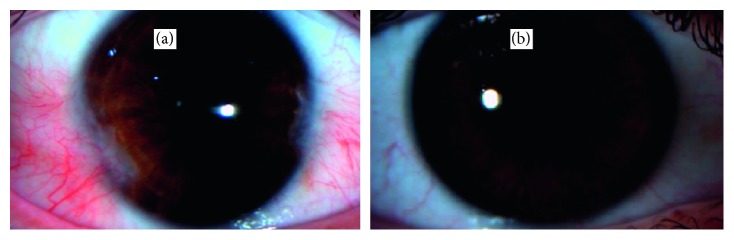
External photograph of case 1 of double-headed pterygia before (a) and 1 year after (b) surgery.

**Figure 2:**
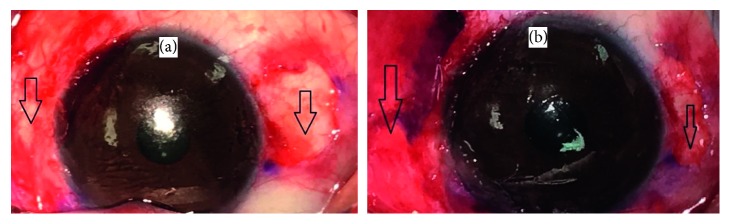
External photograph of case 3 of double-headed pterygium surgery. (a) The conjunctival defects (arrow) after double-headed pterygia excision. (b) The conjunctival autograft (arrow).

Primary outcome measures included the recurrence rate and cosmetic grading at 1-year follow-up compared to baseline (Figures 1 and 2).

## 3. Results

This study included 15 eyes of 15 patients. The mean age of all patients was 36.92 ± 10.8 years. There were 9 men (60%) and 6 women (40%). Out of 30 pterygia from 15 eyes, grade I, II, and III pterygium was found in 9 pterygia (30%), 18 pterygia (60%), and 3 pterygia (10%), respectively. The mean operative time was 32 ± 4.1 minutes. The mean postoperative follow-up was 16 ± 3.4 months. The mean tear breakup time (BUT) improved significantly from 9 ± 1.5 seconds preoperatively to 12.2 ± 2.3 seconds at 12-month follow-up (*P* value = 0.03), while the mean Schirmer's test I improved from 11.6 ± 5 mm preoperatively to 13.6 ± 4.4 mm at 12-month follow-up without a significant change. The mean size of nasal, temporal, and total conjunctival defects was 7.9 ± 1.2, 6.5 ± 1.1, and 13.6 ± 1.5 mm^2^, respectively. Recurrence was not seen in any cases. Regarding postoperative cosmetic grading, grade 1 was found in 12 eyes (80%) and grade 2 was found in 3 eyes (20%). None of the cases showed conjunctival scarring or fibrosis at the conjunctival donor area. The preoperative Sim K astigmatism at the central 3 mm and BCVA were 3.05 ± 1.5 D and 0.64 ± 0.26 logMAR, which improved significantly to 1.15 ± 0.84 D and 0.26 ± 0.18 logMAR at 12-month follow-up postoperatively (*P* value = 0.04 and 0.02), respectively (Table 1). Graft edema and subconjunctival hemorrhage were reported in 7 pterygia (23%) and 8 pterygia (27%), respectively. None of cases showed associated systemic diseases (Table 1).

## 4. Discussion

A perfect pterygium surgery should achieve three principal goals: a low or no recurrence rate, minimal complications, and good cosmetic appearance [20].

The treatment of pterygium includes different surgical approaches such as simple excision, the bare sclera technique, the pterygium extended removal followed by extended conjunctival transplant (P.E.R.F.E.C.T) technique, and lamellar keratoplasty, besides different covering tissues such as sliding conjunctival flaps, conjunctival or limbal autografts, and amniotic membrane transplants and the use of different adjuvant treatments such as β-irradiation, mitomycin, and anti-VEGF [16, 21, 22].

Different surgical approaches for treatment of double-headed pterygia were conducted.

In our cohort for treatment of double-headed pterygia, we conducted simultaneous nasal and temporal pterygia excision using vertical SCG achieving 0% recurrence and good cosmetic success (grades 1and 2).

In the previous publications on double-headed pterygia, recurrence rates ranged from 0% to 35%, while the number of operated eyes ranged from 7 to 87 eyes.

All studies including this cohort defined recurrence as fibrovascular tissue invading the cornea over the limbus except the study of Yeung et al. which defined it as invasion 1 mm over the limbus, while the study of Duman and Kosker accepted recurrence as fibrovascular proliferation more than 0.5 mm over the cornea [16, 23–34].

Previous studies conducted simultaneous excision of nasal and temporal pterygia except 2 studies that undertook sequential procedures with minimal interval between nasal and temporal pterygia excision of 3 and 6 months, which are Yeung et al. and Hirst and Smallcombe studies, respectively. Hirst and Smallcombe stated that the staged approach permitted the surgeon and patient to be sure of the first surgery outcome, and besides, the delay of the second pterygium removal does not carry any significant risk to the patient [16, 30]. We believe that the simultaneous approach decreases the burden of the staged approach on the patients which includes longer treatment duration, two separate surgeries, and delayed surgical outcome.

Few studies undertook the SCG technique: both Maheshwari and Lee et al. split conjunctival grafts horizontally, while Duman and Kosker and Kodavoor et al. bisected them vertically. Kodavoor et al. did not maintain limbus-limbus orientation of the conjunctival graft [25, 27, 33].

In our study, we adopted vertical SCG technique maintaining the limbus-limbus orientation of the graft which gives an advantage of including the limbal tissue in the conjunctival graft for both nasal and temporal sides, restoring the barrier effect of the limbus.

None of our cases showed conjunctival scar or fibrosis at the conjunctival donor area. Keeping Tenon's capsule intact and closing the donor site minimize adverse postoperative reactions in the donor area [35, 36].

In our study, we recorded significant improvement in BUT results, while Schirmer's test results did not significantly change after the operations. It may be because the pterygium had no effect on the aqueous tear film. The increase in goblet cell density and normal mucus fern pattern may cause some improvement in postoperative tear breakup time [37].

Due to the increase in corneal involvement in double-headed pterygium, the induced astigmatism is higher which resulted from an alteration in the tear film, causing an apparent flattening of the normal corneal curvature [38]. Our study showed that the astigmatism decreased and BCVA improved significantly following double-headed pterygia excision. According to our knowledge, none of the previous reports on double-headed pterygia commented on this point. Our results agreed with earlier reports which concluded that excision of pterygium leads to statistically significant reduction in astigmatism, which improves vision significantly [39, 40].

Applying glue instead of sutures for the conjunctival graft attachment in pterygium surgery reduces the postoperative inflammation, pain, foreign-body sensation, and the time of surgery, also avoids suture-related complications, and hastens postoperative recovery but carries the risk of viral transmission and allergic reaction besides rare reports about immune system and cardiac disorders [41, 42].

In this study, we used the fibrin sealant (Tisseel) to attach conjunctival grafts to the sclera and close the conjunctiva at the donor area. This would explain the shorter operative time and is considered as a contributing factor in reducing postoperative fibrosis at the donor area.

The main limitation of this study is the small patient cohort size.

We think a prospective head-to-head comparison between horizontal and vertical split conjunctival grafts in treating primary double-headed pterygia would be beneficial in deciding which technique is superior.

## 5. Conclusion

Vertical split conjunctival autograft using fibrin glue is an effective surgical technique for the treatment of primary double-headed pterygia achieving zero recurrence rate and good cosmetic appearance up to one year in our study sample.

## Figures and Tables

**Table 1:** Summary of demographic and clinical results.

Variable	Result (mean ± SD)
Age (years)	36.9 ± 10.8
Gender, total (%)	
** **Men	9 (60%)
** **Women	6 (40%)
Pterygium grade, total (%) (pterygia)	
** **Grade I	9 (30%)
** **Grade II	18 (60%)
** **Grade III	3 (10%)
Pterygium morphology, total (%) (pterygia)	
** **Atrophic	8 (26.7%)
** **Intermediate	14 (46.6%)
** **Fleshy	8 (26.7%)
Pterygium size (mm)^*∗*^	2 ± 0.7
Follow-up period (months)	16 ± 3.4
Operative time (minutes)	32 ± 4.1
Conjunctival defects/conjunctival grafts (mm^2^)^*∗∗*^	
** **Nasal	7.9 ± 1.2
** **Temporal	6.5 ± 1.1
** **Total	13.6 ± 1.5
BCVA (logMAR)	
** **Preoperative	0.64 ± 0.26
** **12-month follow–up	0.26 ± 0.18
Sim K astigmatism (diopters)	
** **Preoperative	3.05 ± 1.5
** **12-month follow-up	1.15 ± 0.84
BUT (seconds)	
** **Preoperative	9 ± 1.5
** **12-month follow-up	12.2 ± 2.3
Schirmer's test I (mm)	
** **Preoperative	11.6 ± 5
** **12-month follow-up	13.6 ± 4.4
Recurrence, total (%)	Zero (0%)
Cosmetic grading, total (%)	
** **Grade 1	12 (80%)
** **Grade 2	3 (20%)
Postoperative complication, total (%)	
** **Graft edema	7 pterygia (23%)
** **Subconjunctival hemorrhage	8 pterygia (23%)

^*∗*^Horizontal length of the pterygium from the limbus to the head (mm). ^*∗∗*^The size of conjunctival grafts equal to the size of conjunctival defects.

## Data Availability

No data will be available.

## Abbreviations

BCVA: Best corrected visual acuity
BUT: Breakup time
AMT: Amniotic membrane transplant
CAG: Conjunctival autograft
CRA: Conjunctival rotational autograft
MMC: Mitomycin C
SCG: Split conjunctival graft
LogMAR: Logarithm of the minimum angle of resolution
D: Diopter
Sim K: Simulated keratometry
P.E.R.F.E.C.T: Pterygium extended removal followed by extended conjunctival transplant.
